# TiRobot-assisted percutaneous kyphoplasty in the management of multilevel (more than three levels) osteoporotic vertebral compression fracture

**DOI:** 10.1007/s00264-022-05580-1

**Published:** 2022-09-14

**Authors:** Shu Lin, Liu-yi Tang, Fei Wang, Xin-wei Yuan, Jiang Hu, Wei-min Liang

**Affiliations:** Department of Orthopaedics, Sichuan Provincial People’s Hospital, University of Electronic Science and Technology of China, No. 32 West Second Section, First Ring Road, Chengdu, 610072 China

**Keywords:** Osteoporotic vertebral compression fracture, Percutaneous vertebroplasty, Multilevel, Bone cement distribution, Radiation exposure

## Abstract

**Purpose:**

To compare the effectiveness of TiRobot-assisted kyphoplasty with that of the traditional fluoroscopy-assisted approach in treating multilevel osteoporotic vertebral compression fractures.

**Methods:**

In this retrospective study, we collected data from 71 patients (TiRobot-assisted group, *n* = 39; fluoroscopy-assisted group, *n* = 32) with multilevel osteoporotic vertebral compression fracture treated with unilateral traditional TiRobot-assisted or fluoroscopy-assisted percutaneous kyphoplasty. The operative time, infusion volume, length of stay (LOS), hospital expenses, visual analog scale (VAS), Oswestry Disability Index (ODI), radiation exposure, puncture deviation, anterior height of diseased vertebrae, local kyphotic angle, bone cement distribution, and bone cement leakage were compared between the TiRobot- and fluoroscopy-assisted groups.

**Results:**

Of the 257 treated vertebrae, the average amount of bone cement injected in the TiRobot-assisted (142 vertebrae) and fluoroscopy-assisted (115 vertebrae) groups was 4.6 mL and 4.5 mL, respectively. The VAS score was significantly lower in the TiRobot-assisted group at 24 hours post-operatively (*p* = 0.006). The X-ray frequency was 34.7 times in the TiRobot-assisted group and 51.7 times in the fluoroscopy-assisted group (*p* < 0.001). In addition to the operative time, cumulative radiation dose for the surgeon and patient was significantly lower in the TiRobot-assisted group. The hospital expenses of the TiRobot-assisted group were significantly higher (*p* < 0.001). The puncture deviation and bone cement distribution were better in the TiRobot-assisted group (*p* < 0.001). Bone cement leakage was found in 18 and 29 cases in the TiRobot- and fluoroscopy-assisted groups, respectively (*p* = 0.010). One patient in the fluoroscopy-assisted group experienced radiculopathy due to a misplaced puncture but recovered in three months. No radiculopathy was observed in the TiRobot-assisted group.

**Conclusions:**

TiRobot-assisted percutaneous multilevel kyphoplasty is more accurate and has smaller radiometry, a more uniform bone cement distribution, and lower bone cement leakage. This method was therefore accurate and safe.

## Introduction

Osteoporosis changes bone tissue structure, increasing bone fragility and fracture risk. Low back pain is one of the main clinical manifestations. In recent years, osteoporotic vertebral compression fracture (OVCF) incidence has increased in older adults. Due to insufficient anterior vertebral height and spinal deformities, OVCF often leads to low back pain, kyphosis, reduced pulmonary function, abdominal and thoracic content restriction, impaired mobility, and depression [1, 2]. The traditional conservative treatment methods mainly include anti-osteoporosis treatment and long-term bed rest immobilisation, potentially leading to bedsores, oedema, pneumonia, urinary tract infection, or death. Percutaneous vertebroplasty was first introduced by Galibert and Deramend in 1984 in France for treating haemangiomas [3]. Percutaneous vertebroplasty is a direct injection of bone cement into the vertebral body through the vertebral arch’s pedicle. It has the advantages of a short operation time, quick symptom improvement, and low cost, with 75–90% of elderly patients with thoracolumbar osteoporotic fractures experiencing significant pain relief. However, there was no significant effect on vertebral height recovery.

Percutaneous kyphoplasty (PKP) is a minimally invasive surgical technique that corrects kyphosis secondary to collapsed vertebral bodies using a balloon which was first performed in 1998 [4]. The advantage of the bilateral approach is the symmetrical and uniform bone cement distribution in the vertebral body. The cement, inserted across the midline, significantly enhances the stiffness of both vertebral bodies. Reduction of the amount of bone cement remains the most crucial factor to reduce the incidence of bone cement leakage and pulmonary embolism [5, 6]. Therefore, unilateral injection may be the best choice for multilevel vertebral fractures.

With an increasing number of treated vertebral bodies, there are other issues such as puncture deviation, uneven bone cement distribution, and radiation exposure. An accurate percutaneous puncture technique is key to preventing complications in most PKP procedures. Orthopaedic robotic systems are a derivative of navigation technology, which allow for accurate positioning and improved operative convenience [7]. In traditional fluoroscopy, it is difficult to control the puncturing point and angle. The working sleeve end cannot reach the ideal position, resulting in poor cement distribution. When a robot designs the surgical puncture route, the puncture angle can be appropriately increased, and the guide pin can be inserted into the vertebral body all at once through the guidance of the robot arm, such that the end of the working channel is as close as possible to the vertebral body’s midline.

There is no research on treating multi-segmental osteoporotic fractures with an orthopaedic robot. Since September 2017, our department has been using the third generation of the “Tianji” orthopaedic robot (TiRobot; China Food and Drug Administration approval in 2016, developed by Beijing Jishuitan Hospital and Beijing TINAVI Medical Technology Co., Ltd.). This study compared the effectiveness of TiRobot-assisted kyphoplasty with the traditional fluoroscopy-assisted approach in treating multilevel OVCF.

## Methods

### Patient selection

Patients were selected using the following inclusion criteria: (i) painful multilevel (more than three levels) OVCFs, (ii) bone mineral density T-scores <  − 2.5, (iii) bone marrow oedema in the short time inversion recovery of magnetic resonance imaging sequence; (iv) absence of radiological neural compression evidence.

Exclusion criteria were (i) neurological deficits, (ii) tumour or metastasis confirmed by pre-operative examination and post-operative pathology, (iii) intolerance to anaesthesia, and (iv) refractory bleeding disorders.

### Clinical data

This retrospective chart review included 71 patients who met the selection criteria from January 2018 to January 2021, receiving unilateral PKP for thoracolumbar OVCF. The patients were divided into the TiRobot-assisted (39 cases) and fluoroscopy-assisted groups (32 cases). TiRobot- or fluoroscopy-assisted treatment was chosen by patients after the details of the operative procedure were explained by the surgeons. All participants provided written informed consent before enrolment. The hospital’s institutional review board approved this study.

### Surgical procedures

After general anaesthesia, the patients were placed in the prone position. Three senior surgeons performed all interventions in the two groups. PKP was performed with a unilateral transpedicular approach using PKP apparatus (Shanghai Kinetic Medical Co., LTD.) and bone cement (Osteopal V, Germany Heraeus Medical).

### Fluoroscopy-assisted group

When the needle entry point was pinpointed by fluoroscopy of the C-arm machine, an incision was made deep into the fascia. The C-arm machine was used to locate the compressed vertebral body and mark the transpedicular puncture needlepoint. A unilateral pedicle puncture was placed at the posterior third of the vertebral body. After the puncture needle’s core was pulled out, a biopsy was taken through the puncture channel, and a reamer was used to make a hole in the vertebra, through which a balloon was inserted to create a void. Subsequently, the bone cement was injected into the compressed vertebral body. Cement injection was terminated when the vertebral body was filled or there was fluoroscopic evidence of epidural, venous, or transcend plate cement extravasation or extrusion into the vertebral body’s posterior quarter was adequate. After the bone cement outside the body was completely hardened, the needle was pulled out of the sleeve to prevent tailing. The exact surgical procedure was applied to the other compressed vertebral body.

### TiRobot group

**Fig. 1:**
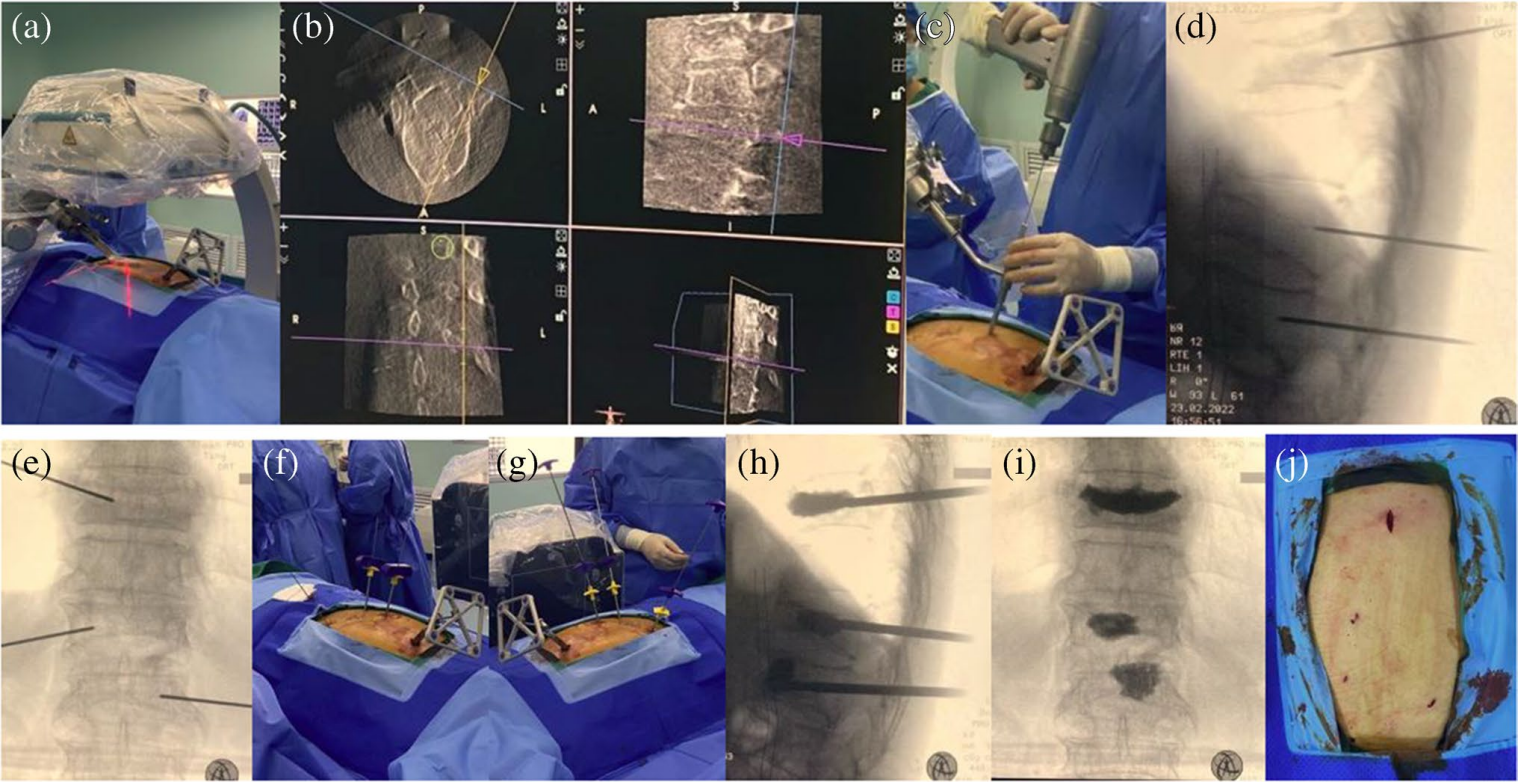
The TiRobot-assisted procedure in a 73-year-old female patient with T10-L1 vertebral compression fracture: (**a**) Robot registration and scanning intra-operative imaging; (**b**) trajectory planning in robot workstation; (**c**) inserting the guidewire with robot-assisted; (**d**), (**e**) intra-operative X-ray film; (**f**) inserting the sleeve; (**g**) cement injection; (**h**), (**i**) intra-operative X-ray film; (**j**) incision

After locating the compressed vertebral bodies, the percutaneous reference tracker was placed in the spinous process superior to the compressed vertebral bodies. A robotic arm held the calibrator on the skin surface of the compressed vertebral bodies, and three-dimensional images were acquired by C-arm scanning, following which the image data was transferred to the robot workstation. Subsequently, we carried out the surgical planning, gave the command, and manipulated the robot arm to the designated position. The sleeve was placed into the mechanical arm’s screw guider. A small incision was made on the skin, and the K-wire (1.5 mm) was drilled into the vertebrae (3.5 cm in depth) through the sleeve. The puncture needle was then propelled by a hammer through the K-wire. The biopsy, balloon dilatation, and cement injection procedures were identical to those of the fluoroscopy-assisted group. The typical procedure performed by the TiRobot is shown in Fig. 1.

### Post-operative care

At six hours post-operatively, the patients were allowed to walk with waist support. All the patients were discharged within two days post-operatively and were required to wear a waist support for four weeks with conservative bisphosphonates, calcitonin, and vitamin D supplementation treatment.

### Data collection

The operative time, infusion volume, length of stay (LOS), and hospital expenses were recorded post-operatively. The visual analog scale (VAS) and Oswestry Disability Index (ODI) were recorded pre-operatively and at 24 h, one month, and one year post-operatively. The patient and surgeon radiation exposures were recorded, including the X-ray frequency.

The patients’ cumulative radiation time and exposure were recorded from the direct output of the C-arm (ARCADIS Orbic 3D system during the operation, provided by Siemens, Germany). The surgeons’ cumulative radiation exposure was recorded using a dosimeter (Sichuan Hongjinda Health Technology Service Co., Ltd.). The dosimeter was attached to the surgeon’s chest outside the lead apron and read by a blinded independent radiologist. A radiographic assessment was recorded, including puncture deviation, bone cement distribution, bone cement leakage, diseased vertebrae anterior height, and local kyphotic angle pre-operatively and at 24 h, one month, and one year post-operatively. Puncture deviation was evaluated according to the modified method of the Gertzbein and Robbins scale [8] (grade A, puncture without a breach of the cortical layer of the vertebral body or pedicle; grade B, cortical breach of < 2 mm; grade C, cortical breach ≥ 2 mm but < 4 mm; grade D, cortical breach ≥ 4 mm but < 6 mm; and grade E, cortical breach ≥ 6 mm). The slice with the most significant deviation from the puncture was chosen for grading.

**Fig. 2:**
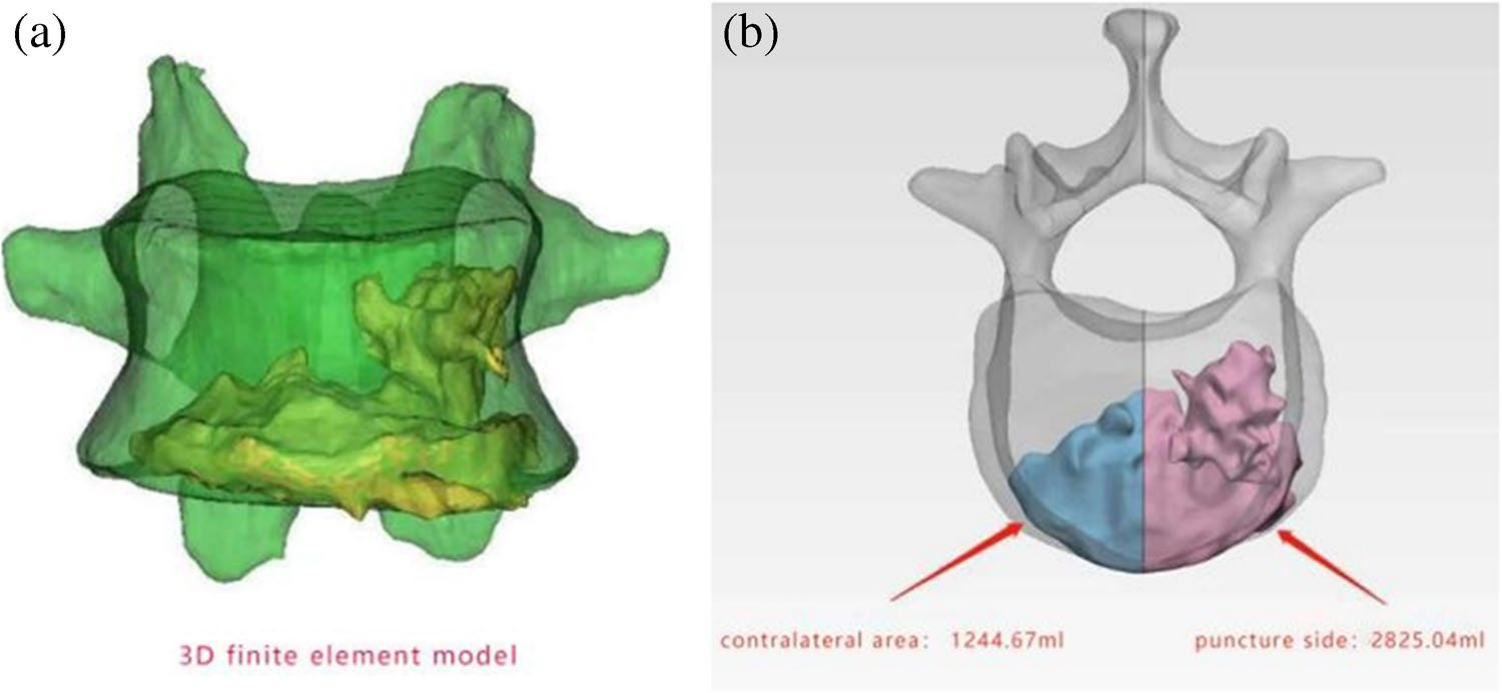
(**a**) 3D finite element model of the post-operative vertebral body. (**b**) Infusion volume bone cement was calculated (puncture side 2825.04 mL; contralateral area 1244.67 mL)

The bone cement distribution was evaluated according to the 3D finite element model of the post-operative vertebral body (Fig. 2). After reconstruction, the vertebral body was equally divided into two parts (puncture side and contralateral area) through the sagittal plane. The bone cement volume in the vertebral body was then calculated. The bone cement leakage rate was assessed by computed tomography one day post-operatively and included the intervertebral, lateral, anterior, and posterior spaces, as well as paravertebral intravascular leakage.

Two senior spine surgeons who did not participate in the operation and blinded to the treatment group evaluated the imaging indexes. Inconsistencies in the results were discussed with the first corresponding author before establishing the final evaluation.

### Statistical analysis

SPSS 19.0 (SPSS Inc., Chicago, IL, USA) was used for statistical analysis. Measurement data were presented as mean ± standard deviations and compared with the *t*-test. The Mann–Whitney *U* test was used to compare continuous variables that were not normally distributed and presented as medians and interquartile ranges. Enumeration data were presented as percentages and compared with a chi-squared test. Statistical significance was set at *p* < 0.05.

## Results

### Patient demographics

Thirty-nine patients (8 men and 31 women; age, 58–86 years; bone mineral density, − 4.6 to − 1.5; BMI, 17.5–32.3 kg/m^2^) were treated with a TiRobot-assisted technique. We had injected 142 vertebrae in those 39 patients from January 2018 to January 2021 (three levels in 23 patients, four in 9 patients, five in 5 patients, and six in 2 patients). Fracture segments included T7 (2 cases), T8 (3 cases), T9 (5 cases), T10 (12 cases), T11 (21 cases), T12 (23 cases), L1 (23 cases), L2 (17 cases), L3 (16 cases), L4 (12 cases), and L5 (8 cases). The number of vertebrae per case was 3.6 in the TiRobot-assisted group. Thirty-two patients had undergone the fluoroscopy-assisted technique from January 2018 to January 2021 (6 men and 26 women; age, 63–85 years; bone mineral density, − 4.3 to − 2.0; BMI, 16.9–31.5 kg/m^2^). Altogether, 115 vertebrae were injected in the 32 patients (three levels in 20 patients, four in 7 patients, five in 3 patients, and six in 2 patients). Fracture segments included T7 (0 cases), T8 (3 cases), T9 (5 cases), T10 (11 cases), T11 (16 cases), T12 (22 cases), L1 (20 cases), L2 (14 cases), L3 (12 cases), L4 (6 cases), and L5 (6 cases). The number of vertebrae per case was 3.5 in the fluoroscopy-assisted group.

The average amount of bone cement injected was 4.65 mL in the TiRobot-assisted group and 4.53 mL in the fluoroscopy-assisted group (*t* = 1.598, *p* = 0.111). There was no statistically significant difference in age, sex, BMI, bone mineral density, number of levels injected, frequency of level injected, vertebrae per case, and the average amount of bone cement injected (Table 1).

**Table 1:** Patient demographics

Characteristic	TiRobot-assisted group (39 cases)	Fluoroscopy-assisted group (32 cases)	Test statistic	*p*-value
Age (years, mean ± *SD*)	71.538 ± 6.348	72.062 ± 6.211	− 0.349	0.728
Sex (male/female, cases)	8/31	6/26	0.035	0.853
Mean BMI (kg/m^2^, mean ± *SD*)	22.897 ± 3.615	23.655 ± 3.635	− 0.876	0.384
Bone mineral density	− 3.364 ± − 0.605	− 3.240 ± − 0.599	− 0.859	0.393
Number of levels injected				
3 levels	23	20	− 0.291	0.771
4 levels	9	7		
5 levels	5	3		
6 levels	2	2		
Frequency of level injected	142/3.6	115/3.5		
T7	2	0	− 0.584	0.559
T8	3	3		
T9	5	5		
T10	12	11		
T11	21	16		
T12	23	22		
L1	23	20		
L2	17	14		
L3	16	12		
L4	12	6		
L5	8	6		
Vertebrae per case	3.641 ± 0.902	3.593 ± 0.910	0.219	0.828
Infusion volume (mL)	4.649 ± 0.578	4.527 ± 0.644	1.598	0.111

*SD* standard deviation, *BMI* body mass index

### Comparison of pain and functional efficacy between the two groups

The VAS score was significantly lower in the TiRobot-assisted group than in the fluoroscopy-assisted group at 24 h post-operatively (2.711 ± 0.964 vs. 3.052 ± 1.007; *p* = 0.006). However, there were no differences in VAS and ODI scores between the two groups at the other time points (*p* > 0.05, Table 2).

**Table 2:** Comparison of pain and functional efficacy

	TiRobot-assisted group (39 cases)	Fluoroscopy-assisted group (32 cases)	Test statistic	*p*-value
VAS score				
Preoperative	5.993 ± 1.107	6.069 ± 0.875	− 0.604	0.546
24 h postoperative	2.711 ± 0.964	3.052 ± 1.007	− 2.762	0.006
1 month postoperative	2.126 ± 0.650	2.052 ± 0.646	0.916	0.360
1 year postoperative	1.647 ± 0.976	1.582 ± 1.034	0.519	0.604
ODI				
Preoperative	76.253 ± 7.041	75.339 ± 7.219	1.023	0.307
24 h postoperative	36.831 ± 10.226	37.252 ± 10.015	− 0.331	0.741
1 month postoperative	29.831 ± 4.826	30.226 ± 4.692	− 0.661	0.509
1 year postoperative	27.859 ± 4.998	27.373 ± 4.621	0.801	0.424

*VAS* visual analog scale, *ODI* Oswestry Disability Index

### Comparison of the local kyphotic angle and the anterior vertebral height between the two groups

There were no differences in the anterior vertebrae height and the local kyphotic angle between the two groups at each time point (*p* > 0.05, Table 3).

**Table 3:** Comparison of pain and functional efficacy

	TiRobot-assisted group (142 vertebrae)	Fluoroscopy-assisted group (115 vertebrae)	Test statistic	*p*-value
Anterior height of diseased vertebrae (mm)				
Preoperative	16.080 ± 4.116	16.800 ± 3.593	− 1.466	0.144
24-h post-surgery	21.035 ± 4.041	21.156 ± 4.855	− 0.214	0.830
1 month postoperative	21.183 ± 4.104	21.756 ± 4.908	− 1.001	0.318
1 year postoperative	21.176 ± 4.069	21.460 ± 5.295	− 0.474	0.626
Local kyphotic angle (°)				
Preoperative	17.363 ± 5.647	17.421 ± 4.416	− 0.089	0.929
24 h postoperative	12.601 ± 3.730	11.912 ± 3.775	1.463	0.145
1 month postoperative	12.216 ± 3.209	12.464 ± 3.464	− 0.594	0.553
1 year postoperative	12.209 ± 3.212	12.517 ± 3.607	− 0.722	0.471

### Radiation exposure and clinical data

The X-ray frequency during the whole procedure was 34.7 times in the TiRobot-assisted group and 51.7 times in the fluoroscopy-assisted group (*p* < 0.001). The surgeons’ cumulative radiation dose was significantly lower in the TiRobot-assisted group (31.410 ± 6.020 μSv) than in the fluoroscopy-assisted group (49.750 ± 10.188 μSv; *p* < 0.001). The patients’ cumulative radiation dose was significantly lower in the TiRobot-assisted group (426.282 ± 73.842 cGycm^2^) than in the fluoroscopy-assisted group (582.781 ± 82.220 cGycm^2^; *p* < 0.001). Radiation exposure is shown in Table 4.

**Table 4:** Radiation exposure and clinical data

	TiRobot-assisted group (39 cases)	Fluoroscopy-assisted group (32 cases)	Test statistic	*p*-value
Patient radiation exposure (cGycm^2^, mean ± *SD*)	426.282 ± 73.842	582.781 ± 82.220	− 8.442	< 0.001
Surgeon radiation exposure (μSv, mean ± *SD*)	31.410 ± 6.020	49.750 ± 10.188	− 9.422	< 0.001
X-ray frequency (freq)	34.769 ± 8.222	51.781 ± 9.255	− 8.197	< 0.001

*SD* standard deviation

### Comparison of clinical data between the two groups

The operative time of the TiRobot-assisted group (47.153 ± 7.128 min) was significantly shorter than that of the fluoroscopy-assisted group (57.656 ± 5.445 min; *p* < 0.001). LOS was not significantly different between the two groups (*p* = 0.158). The hospital expenses were significantly higher in the TiRobot-assisted group (58,676.565 ± 5651.421 yuan) than in the fluoroscopy-assisted group (46,679.250 ± 4817.461 yuan; *p* < 0.001; Table 5).

**Table 5:** Comparison of clinic data

	TiRobot-assisted group (39 cases)	Fluoroscopy-assisted group (32 cases)	Test statistic	*p*-value
Operative time (min)	47.153 ± 7.128	57.656 ± 5.445	− 6.851	< 0.001
Length of stay (days)	8.128 ± 2.015	7.406 ± 2.241	1.428	0.158
Hospital expenses (yuan)	58,676.565 ± 5651.421	46,679.250 ± 4817.461	9.503	< 0.001

### Puncture deviation

In the TiRobot-assisted group, 91.5% of the screws had an optimal puncture (grade A). The remaining punctures were of grades B (6.3%), C (2.1%), and D (0%). In the fluoroscopy-assisted group, 66.9% of the screws had an optimal puncture (grade A). The remaining punctures were of grades B (13.0%), C (12.1%), and D (7.8%). Puncture deviation was better in the TiRobot-assisted group than in the fluoroscopy-assisted group (*p* = 0.000). Puncture deviation is shown in Table 6.

**Table 6:** Puncture deviation

	Deviation of puncture				Test statistic	*p*-value
	A	B	C	D		
TiRobot-assisted group	130	9	3	0	− 5.141	< 0.001
Fluoroscopy-assisted group	77	15	14	9		

One patient in the fluoroscopy-assisted group experienced painful radiculopathy due to a misplaced puncture but recovered in three months. In contrast, no cases of painful radiculopathy were observed in the TiRobot-assisted group (*p* > 0.999).

### *Bone cement distribution*

The fluoroscopy-assisted group had significantly more bone cement on the puncture side than the TiRobot-assisted group (*p* < 0.001), which also showed better bone cement distribution than the fluoroscopy-assisted group (*p* < 0.001). Bone cement distribution is shown in Table 7.

**Table 7:** Bone cement distribution

	Puncture side	Contralateral area	Statistical value	*p*-value
TiRobot-assisted group	2822.514 ± 715.47	1826.936 ± 556.095	178.284	< 0.001
Fluoroscopy-assisted group	3341.400 ± 754.709	1186.034 ± 500.301		

### Comparison of post-operative bone cement leakage between groups

Bone cement leakage was found in 18 cases in the TiRobot group (5, 3, and 7 cases in the intervertebral, lateral, and anterior spaces, respectively, and 3 cases of paravertebral intravascular leakage) and in 29 cases in the fluoroscopy-assisted group (12, 8, 5, and 1 cases in the intervertebral, lateral, anterior, and posterior spaces, and 3 cases of paravertebral intravascular leakage); these rates were significantly different between groups (*p* = 0.010). The leakage was not accompanied by nerve irritation. No other complications appeared, such as infection and pulmonary embolism.

## Discussion

Although there is little literature on multilevel osteoporotic spine fractures treated with PKP, such cases are not uncommon in China [9]. Patients with multi-segmental osteoporotic fractures often present with kyphosis and frontal deformity due to right–left asymmetrical compression, increasing the difficulty of percutaneous puncture. Therefore, an accurate puncture is key to treating multilevel OVCF with PKP.

The orthopaedic robot system is a derivative of navigation technology, which allows for accurate positioning and increased surgical convenience. The accuracy of TiRobot implants in the thoracolumbar spine is well recognised [10–14]. Our results showed that the puncture deviation of the robot-assisted group was better than that of the traditional management group, which indicated that robot-assisted PKP could improve puncture accuracy and reduce the risk of neurovascular injury.

This study analysed cement distribution on both sides of the vertebral body using the finite element method. The cement reached the non-punctured side more quickly and had a more uniform distribution in the TiRobot group. In the traditional fluoroscopy group, the bone cement was mainly concentrated on the punctured side, which may result in vertebral body pressure imbalance.

In the traditional fluoroscopy, the puncturing point often cannot be accurately located at the first attempt and needs to be adjusted multiple times, damaging the surrounding joint capsule [15]. Similarly, after entering the pedicle, the inclination angle may be too small and require some adjustment, resulting in pedicle injury. This could likely explain the better VAS score of the TiRobot group at 24 h post-operatively. However, there was no significant difference between the groups at six months post-operatively.

For multi-segmental OVCF, conventional fluoroscopy-guided PKP requires multiple punctures and repeated fluoroscopy, prolonging the operative time and potentially increasing the radiation exposure to doctors and patients. In this study, the radiation exposure was significantly lower in the TiRobot group. Moreover, robotic assistance reduced patients’ radiation exposure and the puncture, fluoroscopy, and operative times.

Precise and individual minimally invasive surgery has become the new trend. Spine surgeons favour orthopaedic surgical robots because of their advantages such as high stability, high precision, increased surgical simplicity and convenience, and short learning curve [16]. Vardiman et al. [17] reported that the personal surgical experience has little impact on accuracy of pedicle screw placement with robotic assistance, indicating that the robot-assisted learning curve is shorter and can be quickly mastered by inexperienced doctors. Due to the high precision of the orthopaedic robot system, surgeons are more confident in performing multilevel PKP.

### Limitations

This study only presents a retrospective single-centre experience, as there was a lack of multicentre-controlled studies. The number of cases needs to be increased to enhance the study conclusions’ credibility. While the present study’s follow-up period of one year was on par with most of the existing literature, a more extended follow-up might enable us to better understand the long-term effect of this novel surgical treatment. Using a retrospective research method inevitably leads to the loss of some clinical data; therefore, selection bias is possible. A prospective randomised study design combined with extended follow-up and multicentre studies is needed.

## Conclusion

TiRobot-assisted percutaneous multilevel kyphoplasty is more accurate than the traditional fluoroscopy-assisted technique and shows smaller radiometry, more uniform bone cement distribution, and lower bone cement leakage rate. This method was therefore accurate and safe.
